# Hemagophagocytic lymphohistiocytosis associated with recurrent Babesiosis with Lyme disease co-infection in an immunocompromised host on anti-CD20 monoclonal antibody therapy: A case report

**DOI:** 10.1016/j.idcr.2022.e01570

**Published:** 2022-07-13

**Authors:** Aleena Zahra, Luis A. Marcos

**Affiliations:** SUNY Stony Brook University Hospital, Department of Medicine, Division of Infectious Diseases, 101 Nicolls Road HSC 16-040, Stony Brook, NY 11794, the United States of America

**Keywords:** Babesiosis, Hemagophagocytic lymphohistiocytosis, Infectious Complication with anti-CD20 Antibody Therapy, Tick-borne disease, Lyme and Babesiosis co-infection

## Abstract

In the Northeastern United States, a nationally notifiable emerging infectious disease caused by a protozoan infecting erythrocytes is endemic. Transmitted by a deer tick, *Ixodes scapularis*, this protozoan, *Babesia microti*, has a complex life cycle including multiple mammalian hosts with humans affected as a dead end reservoir. Although humans are not necessary for the survival of this protozoan, if human erythrocytes are infected by it, especially in a host that is immunocompromised, devastating clinical illness with a significant risk of mortality occurs. Erythrocytic infection of the human host causes many pathogenic changes within the human body, leading to red blood cell destruction and release of pro inflammatory cytokines (3). This pro-inflammatory cascade can very rarely lead to a cycle of further erythrocytic destruction through development of a rare syndrome with high mortality, hemagophagocytic lymphohistocytosis (HLH), which requires early recognition and diagnosis for treatment. This case discusses infection in an immunocompromised host with Babesiosis with complications of HLH and co-infection with Lyme disease requiring multiple diagnostic and therapeutic decisions.

## Highlights

• Human Babesiosis is an emerging infectious disease in the Northeastern United States, particularly in Long Island, NY.
• Babesiosis can lead to a rare syndrome (pro-inflammatory cascade) with high mortality, Hemagophagocytic lymphohistiocytosis.
• HLH may present as a management challenge in Babesiosis, as it requires addition of steroids to anti parasitic therapy.

## Introduction

Babesiosis is an emerging, nationally notifiable infectious disease in the United States, infections are found worldwide with various clades in different worldwide distributions. The predominant cluster in the Northeastern United States is caused by *B microti,* a protozoa of the genus *Babesia* and is transmitted by the tick *I scapularis*. Infection can manifest in a wide range from asymptomatic to severe clinical pathology and can cause significant morbidity and mortality, especially in immunocompromised hosts in whom mortality can reach up to 20 % in hospitalized patients with severe Babesiosis [3], [5]. In hosts with functional or true asplenia or those with functional B cell deficiency, either due to malignancy or anti CD20 monoclonal antibody therapy, disease manifestations are increasingly severe with organ dysfunction and severe laboratory derangements. Rarely, this includes development of Hemagophagocytic Lymphohistiocytosis (HLH); therapy in these individuals requires longer courses of treatment and closer monitoring parameters [1], [2], [5]. Individuals with Babesiosis can also be co-infected with other pathogens carried by *I scapularis* and require screening and treatment, if necessary, for these. This case will discuss Babesiosis infection in an immunocompromised patient and the diagnostic and therapeutic challenges posed.

## Case

A 62-year-old Female presented to a university hospital (UH) in Long Island, NY in early fall with a chief complaint of fatigue for one month duration and one week duration of daily high fevers (Tmax 40.5 °C, reference range 36.1–37.2 °C). Her past medical history was significant for Multiple Sclerosis treated with Ocrelizumab, an anti-CD20 antibody, Babesiosis diagnosed and treated with complete symptom resolution nine months prior to current presentation, Diabetes mellitus type 2, Hypothyroidism, and Lymphocytic colitis. Her social and travel history was relevant for time split between South America (last six months ago, area is not known to be endemic for malaria), a large city in Northeastern US, and at time of presentation, residence at a beachside home on Long Island, NY.

She initially presented to an outside hospital and workup undertaken there revealed anemia, thrombocytopenia, blood smear with intraerythrocytic inclusions, and a peak parasitemia of 1 % and physical exam findings of palpable splenomegaly. Babesiosis was diagnosed and treatment with Azithromycin and Atovaquone was initiated, however anemia, thrombocytopenia, daily high fevers (Tmax 40.5 °C, reference range 36.1–37.2 °C) and parasitemia were persistent for approximately seven days and she was transferred to UH for further evaluation including for plasma exchange transfusion.

**Image 1:**
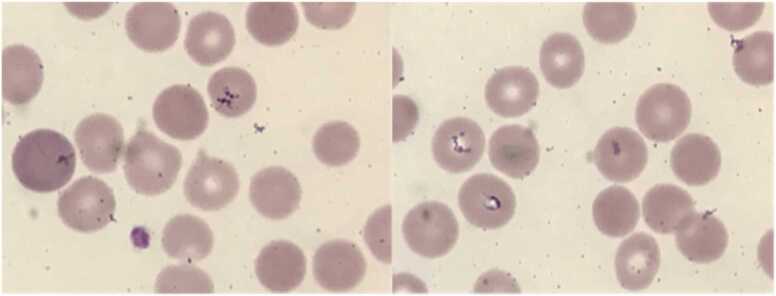
Peripheral blood thin smear, 40 × magnification. Intra-erythrocytic ring inclusions, Maltese cross.

**Image 2:**
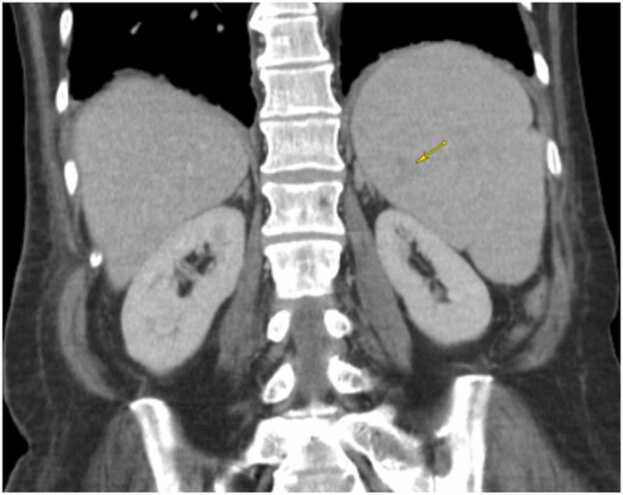
Computer Tomography (CT) Abdomen and Pelvis with intravenous contrast, Coronal view. Faint splenic wedge-shaped posterior ischemia or infarct (yellow arrow) and splenomegaly (left side) measuring 15.7 × 9.2 cm.

At UH she continued to have persistent parasitemia 0.4–1.5 % (reference range 0 %), daily fevers Tmax 38–40.5 °C, worsening hemolytic anemia, leukopenia, thrombocytopenia, indirect hyperbilirubinemia, elevated ferritin and LDH (Table 1). Infectious Disease was consulted. On physical exam vital signs were significant for T 38.5 °C, blood pressure 91/53 mm Hg (reference range < 120/80 mm Hg) and heart rate 120 bpm (reference range 60–120 bpm). She was alert and appeared pale and fatigued, also notable was conjunctival pallor, mild scleral icterus, and palpable splenomegaly, there was no rash, cardiac murmur, diminished lung sounds, or palpable lymphadenopathy. Examination of blood smear revealed intra erythrocytic inclusion and Maltese cross (Image 1). Radiographic imaging included Computed Tomography (CT) abdomen and pelvis with intravenous contrast and abdominal ultrasound, CT revealed faint splenic wedge-shaped posterior ischemia/infarct and splenomegaly 15.7 × 9.2 cm (Image 2). Ultrasound also significant for splenomegaly, 17 cm (reference range < 12 cm).

**Table 1:** Relevant labs trends during hospitalization. Values in red reflect noted abnormalities.

She continued treatment with Azithromycin and Atovaquone and started Doxycycline for Lyme disease co-infection. Exchange transfusion was not undertaken due to low level parasitemia and subjective clinical improvement. Due to persistent laboratory parameter derangements as above, development of Hemagophagocytic Lymphohistiocytosis like syndrome with immune system overactivation was of concern. Hematology was consulted and Prednisone was initiated with improvement in laboratory parameters and patient’s clinical condition. Bone marrow biopsy was deferred at the time by Hematology and empiric treatment was initiated with Prednisone. Patient subsequently defervesced and was discharged to home after ten days hospitalization with a plan of six weeks Azithromycin and Atovaquone for Babesiosis and three weeks of Doxycycline for Lyme co-infection with planned follow up with Infectious Diseases. After discharge she was lost to follow up.

## Discussion

Babesiosis is an emerging infectious disease with *Babesia microti* cases clustered in the Northeastern United States. Disease characteristics are similar to Malaria and associated with significant morbidities. The disease is caused by a protozoa of the Genus *Babesia* and is transmitted by tick vectors, less commonly by blood transfusions, organ transplantation, or congenitally [3]. The incubation period is usually one to four weeks from tick bite, although up to seven weeks has been observed, and can be one week to six months after blood transfusion. Subclinical disease may transform into clinical disease if splenectomy or an immunosuppressive condition, such as malignancy, develops. Babesiosis can lead to significant morbidity, with up to 50 % of patients hospitalized, most often secondary to severe anemia, acute respiratory distress syndrome, congestive heart failure, or renal failure. Mortality is 3–9 % in hospitalized patients and up to 20 % of immunosuppressed patients [3].

Similar to *Plasmodium* spp., *Babesia* spp. infect mammals and lyse host red blood cells, however unlike for malaria, humans are not reservoir hosts and there is no exoerythrocytic stage or hemozoin red blood cell deposit. Babesiosis is transmitted through tick bite of *I scapularis* in the months of May to September, with three quarters of cases from June-August. Nymphal ticks are the primary vector, although adult ticks can still transmit. The incidence of Babesiosis has increased in the past three decades in the Northeastern US and it has been a nationally notifiable disease since January 2011 in the United States. Increased incidence is attributed to increased deer numbers (reforestation, lack of predators), heightened community awareness, and habitation in wooded areas [3].

Pathogenesis of *B microti* infection has been studied in animal models of mice, cattle, and dogs. A proposed model is merozoite release by erythrocytes leads to loss of RBC cell membrane integrity, leading to hemolysis, fever, anemia, jaundice, hemoglobinuria, and renal insufficiency. The splenic macrophages ingest and clear infected erythrocytes, the spleen thus holds a critical role in protection against *Babesia* spp. infection. An additional possible mechanism is *B microti* infection leads to generation of reactive oxygen species and membrane lipid peroxidation, leading to decreased RBC deformability and increasing RBC clearance by splenic macrophages. This may lead to the observed clinical symptoms of anemia, reticulocytosis, increased erythropoiesis, and anemia more prolonged than parasitemia due to the increased clearance by splenic macrophages [2], [3], [4].

These symptoms were also observed in our patient. Although the role of antibody formation in host defense is not clearly understood and in mouse model's antibody formation is not critical, formation of antibody is of critical importance in patients with anti-CD20 antibody treatment. These patients, as with our patient, have increased risk for persistent and relapsing Babesiosis. It is also important to note resolution of parasitemia often coincides with seroconversion [5]. Our patient did have evidence of Babesia IgG Ab seroconversion and this has implications for treatment course as will be discussed.

In addition, CD4+ T cells are central to host immunity, and Interferon gamma is critical to host resistance (from CD4+ T cells for *B microti*). This increases surface MHC II class expression in antigen presenting cells (APCs) and induces upregulated inflammatory cytokine gene expression. The host inflammatory response also includes pyrogenic cytokines (TNFa, IL-6) leading to fever, headache, myalgia (viral like illness) and acute lung injury (severe babesiosis, not observed in our patient). It is also critical to note that from the proposed mechanisms, merozoite release causing erythrocyte damage, and independent generation of reactive oxygen species with subsequent inflammatory pathway activation leads to the observed clinical manifestations. The activation of inflammatory cytokines is the most likely etiology for development of subsequent hemagophagocytic lymphohistiocytosis [1], [2], [5]. Clinical manifestations of Babesiosis are likely due to the pathogenesis mechanisms as discussed above.

Development of severe disease is a significant risk for individuals with asplenia (can be fatal), immunosuppression including following solid organ or stem cell transplant, HIV/AIDS with low CD4, malignancy, chemotherapy, Tumor necrosis alpha (TNF-a) blockade, anti CD20 monoclonal antibody therapy or CD20 depletion (our patient was receiving anti CD20 monoclonal antibody therapy), age > 50 years (our patient is in this group), and neonates. Relapsing disease despite a standard course may be seen in patients with malignancy, particularly B cell lymphoma, anti CD20 therapy, HIV/AIDS, and organ, stem cell transplant. It is important to note relapsing disease occurs few days to one week after discontinuing antibiotics and is less severe than original disease [3], [5]. Our patient presented nine months after completing course of treatment for Babesiosis with complete resolution of symptoms until presentation with increased disease severity compared to prior occurrence, this argues against relapsing disease despite her risk factor of anti CD20 therapy for relapsing disease, and most likely indicates re-infection.

It is also expected that co-infection with other pathogens carried on *I scapularis* can occur, this includes *B burgdorferi*, the causative of Lyme disease*,* and also includes *A phagocytophilum (*Anaplasmosis), *B miyamotoi* and *mayonii*, and Powassan virus*.* Our patient did have Lyme disease co-infection from screening with no clear symptoms of Lyme disease. Based on screening we were able to treat for this with the addition of Doxycycline to her regimen. Our index of suspicion was higher due to known infection with another *I scapularis* carried pathogen. This highlights the importance of screening for other relevant (based on carriers, geographic distribution) of tick-borne diseases following the identification of one in a host.

Treatment and duration for Babesiosis is dependent on severity of disease and co-morbidities. Immunocompetent individuals with parasitemia < 4 % and mild- moderate symptoms may be treated with a seven to ten day course of oral Azithromycin and Atovaquone combination (preferred) or oral Clindamycin and Quinine (preferred in Pregnancy), no hospital admission is usually required. For immunocompromised patients, mild-moderate disease should be treated until no evident parasitemia, and severe disease always warrants hospitalization. Groups considered at high risk for relapsing Babesiosis are those with B cell lymphoma, anti CD20 therapy, malignancy, asplenia, solid organ or stem cell transplant, or HIV/AIDS. In this patient population, treatment must be continued for at least six weeks and should be discontinued only after no parasitemia is observed in thin smear for two consecutive weeks, although relapsing may be seen even with smears and PCR negative in blood stream [6]. Quantified parasitemia is preferred to PCR as PCR is expected to remain positive following clinical cure for up to 27 months in immunosuppressed groups [1], [3].

An additional complicating factor with severe Babesiosis is development of Hemagophagocytic Lymphohistocytosis (HLH). This is a rare but frequently fatal complication. HLH occurs secondary to immune system overactivation leading to engulfment of erythrocytes, platelets, and precursors by macrophages. This disorder may be inherited or acquired secondary to immune homeostasis alteration through infection or hematologic malignancy. Clinical manifestations include a febrile illness with multiple organ involvement including bone marrow (cytopenias), liver, and brain. Laboratory parameters are significant for pancytopenia and high serum ferritin [1], [2], [4], [5]. Our patient met these laboratory parameters and had a persistent febrile illness, she was treated with steroids (Prednisone) with clinical improvement and defervescence and improvement in cytopenias. HLH with Babesiosis has only been reported in a few immunocompromised patients and two immunocompetent patients, it carries high risk of mortality and requires early recognition and treatment. HLH is crucial to consider in patients with severe Babesiosis and elevated inflammatory markers and cytopenias; it presents a diagnostic challenge and alters therapeutic strategy as it requires addition of steroids to anti parasitic therapy.

This case emphasizes several important considerations. Babesiosis is an emerging infectious disease and *B microti* is endemic in the Northeastern United States, it must be considered in cases of febrile illness and cytopenias, especially in summertime. In the immunocompetent host, this infection can be treated with relatively short courses, usually ten days, however in the immunocompromised host, especially with B cell functional or absolute deficiency or functional or true asplenia, the disease can have devastating consequences with multiorgan involvement and carries a risk of mortality up to 20 %.

Here, we presented a patient with a diagnostic challenge of the question of relapse or recurrence and additionally required consideration of persistently elevated inflammatory markers leading to suspicion of, and treatment for concomitant HLH. Based on the proposed pathogenesis for Babesiosis, pro-inflammatory cytokines (TNF alpha, IL-6) are activated through a cascade including CD4+ T cells, interferon gamma, and increased surface MHC II class expression in antigen presenting cells. We hypothesize the activation of these pro-inflammatory cytokines by primary *B microti* infection may lead to immune system overactivation and the associated HLH syndrome. Babesiosis continues to provide a diagnostic and therapeutic challenge and requires further research into pathogenesis, source control, as humans are dead end hosts, accurate diagnosis with consideration of co-morbidities and co-infections, and therapeutics.

## CRediT authorship contribution statement

**Aleena Zahra:** Writing – original draft, Writing – review & editing, data collection. **Luis A Marcos:** Writing – original draft, Writing – review & editing, Supervision.

## Ethical approval

n/a.

## Funding sources

This research did not receive any specific grant from funding agencies in the public, commercial, or not-for-profit sectors.

## Conflict of interest statement

The authors have no conflicts of interest to declare.
